# Supplementary material for: Identification of photoperiod-induced specific miRNAs in the adrenal glands of Sunite sheep (Ovis aries)
Source: Front Vet Sci. 2022 Jul 22;9:888207. doi: 10.3389/fvets.2022.888207 (PMC9354845; doi:10.3389/fvets.2022.888207)
Supplement: Supplementary file 1 [file Data_Sheet_1.ZIP › Supplementary file/miRDeep2 file.zip!/miRDeep2 file/Know_Structure/SPLP42A2/oar-mir-200b.pdf]

miRBase precursor : oar-mir-200b
Total read count : 332
oar-miR-200b read count : 325
remaining reads : 7

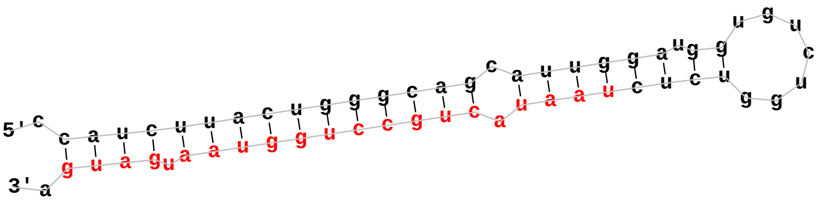

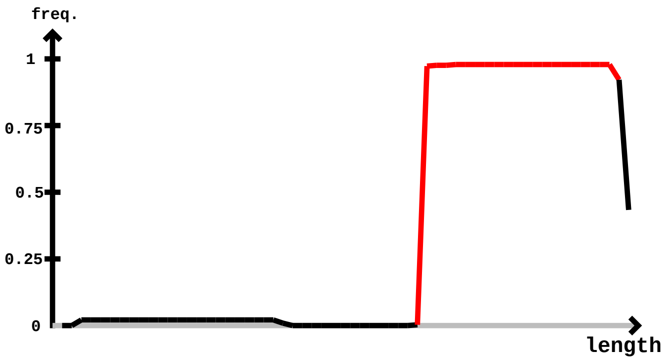


```
                                                        oar-miR-200b
5'- ccaucuuacugggcagcauuggauggugucuggucucuaauacugccugguaaugaugа   -3'    exp
    .(((((((((((((((.((((((.((.......)))))))).))))))))))).)))).    reads      mm          sample
    .caucuuacugggcagcauugg.....................................    4          0           seq
    .caucuuacugggcagcauugga....................................    3          0           seq
    ....................................cuaauacugccugguaaugauga    1          0           seq
    .....................................uaauacugccugguaaugau..    19         0           seq
    .....................................uaauacugccugguaaugaug.    161        0           seq
    .....................................uaauacugccugguaaugauga    142        0           seq
    ......................................aauacugccugguaaugaug.    1          0           seq
    ........................................uacugccugguaaugauga    1          0           seq
```
